# Minimal Self and Timing Disorders in Schizophrenia: A Case Report

**DOI:** 10.3389/fnhum.2018.00132

**Published:** 2018-04-06

**Authors:** Brice Martin, Nicolas Franck, Michel Cermolacce, Jennifer T. Coull, Anne Giersch

**Affiliations:** ^1^Centre Ressource de Réhabilitation Psychosociale et de Remédiation Cognitive, Centre Référent Lyonnais en Réhabilitation et en Remédiation Cognitive Hôpital du Vinatier, Centre National de la Recherche Scientifique UMR 5229, Lyon, France; ^2^Service Universitaire de Psychiatrie, Hôpital Ste Marguerite, Marseille, France; ^3^Laboratoire des Neurosciences Cognitives (UMR 7291), Aix-Marseille Université & Centre National de la Recherche Scientifique, Marseille, France; ^4^Institut National de la Santé et de la Recherche Médicale U1114, Pôle de Psychiatrie, Fédération de Médecine Translationnelle de Strasbourg, Centre Hospitalier Régional Universitaire of Strasbourg, Université de Strasbourg, Paris, France

**Keywords:** self disorders, schizophrenia, timing and time perception, minimal self, hazard function, simultaneity judgment, implicit

## Abstract

For years, phenomenological psychiatry has proposed that distortions of the temporal structure of consciousness contribute to the abnormal experiences described before schizophrenia emerges, and may relate to basic disturbances in consciousness of the self. However, considering that temporality refers mainly to an implicit aspect of our relationship with the world, disturbances in the temporal structure of consciousness remain difficult to access. Nonetheless, previous studies have shown a correlation between self disorders and the automatic ability to expect an event in time, suggesting timing is a key issue for the psychopathology of schizophrenia. Timing disorders may represent a target for cognitive remediation, but this requires that disorders can be demonstrated at an individual level. Since cognitive impairments in patients with schizophrenia are discrete, and there is no standardized timing exploration, we focused on timing impairments suggested to be related to self disorders. We present the case report of AF, a 22 year old man suffering from schizophrenia, with no antipsychotic intake. Although AF shows few positive and negative symptoms and has a normal neurocognitive assessment, he shows a high level of disturbance of Minimal Self Disorders (SDs) (assessed with the EASE scale). Moreover, AF has a rare ability to describe his self and time difficulties. An objective assessment of timing ability (variable foreperiod task) confirmed that AF had temporal impairments similar to those previously described in patients, i.e., a preserved ability to distinguish time intervals, but a difficulty to benefit from the passage of time to expect a visual stimulus. He presents additional difficulties in benefitting from temporal cues and adapting to changes in time delays. The impairments were ample enough to yield significant effects with analyses at the individual level. Although causal relationships between subjective and objective impairments cannot be established, the results show that exploring timing deficits at the individual level is possible in patients with schizophrenia. Besides, the results are consistent with hypotheses relating minimal self disorders (SDs) to timing difficulties. They suggest that both subjective and objective timing investigations should be developed further so that their use at an individual level can be generalized in clinical practice.

## Introduction

Psychiatrists in the field of both phenomenology and experimental psychology have proposed that disturbances of temporality in schizophrenia represent a key issue for psychopathology (Fuchs, 2007; Vogeley and Kupke, 2007). Timing disorders may relate especially to basic disturbances in consciousness of the self i.e., the “minimal self” (Martin et al., 2014; Giersch and Mishara, 2017a). The minimal self is defined by the pre-reflexive “mineness” of conscious experience (Gallagher, 2000). For example, we say “I see a tree” when pointing out a tree to another person. In this sentence, the subject says “I” without thinking about it, as a result of his/her natural first-person perspective. The presence of the “I” signals the sense of “minimal self.” As explained by Stanghellini (2009) “I experience myself as the perspectival origin of my experiences (i.e., perceptions or emotions), actions and thoughts.” Minimal Self disorders (SDs) are considered as trait markers. They have been described in prodromal, as well as chronic stages of schizophrenia (Møller and Husby, 2000; Parnas et al., 2003, 2005; Raballo et al., 2011). They are considered stable, core features of schizophrenia (Lysaker and Lysaker, 2010; Gallese and Ferri, 2013; Nelson et al., 2013b; Hur et al., 2014; Mishara et al., 2014; Nordgaard and Parnas, 2014; Nordgaard et al., 2017), and discriminate the schizophrenia spectrum from bipolar disorders with psychotic experiences and other psychoses (Haug et al., 2012a), or borderline personality (Nelson et al., 2013a). However, descriptions of temporality disorders are rarer. Temporality is the sense of continuity that is inherent to the concept of minimal self: we implicitly think of ourselves as unique beings that are continuous in time. Our sense of self continuity is a given, which we do not naturally question. It is so strong a feeling that it is difficult to imagine its perturbation, making it difficult to access by verbal report. Timing disorders are thus often ignored in clinical settings, despite their potential role in pathophysiology. They have however been demonstrated in group studies, and have been related to minimal self disorders (Martin et al., 2017). Here we check whether it is possible to demonstrate timing disorders at an individual level, which is a critical step toward the integration of such evaluations into clinical practice.

We present a case report of AF because he has the rare ability to describe his time and self disorders (“subjective” timing). We also report experimental results (“objective” timing) analyzed at the single case level in AF, focusing on temporal expectation in the seconds time-range. Complementary results on a simultaneity judgement task in the millisecond range (Martin et al., 2014; Giersch and Mishara, 2017a,b), can be found in Supplementary Materials. We explored implicit timing, which is defined by the processing of information in time independently of any explicit temporal judgement (Coull and Nobre, 2008). This type of timing is very close to the phenomenological description of human time in that it shapes conscious experience rather than representing the contents of time. We favor this approach because explicit duration judgements are characterized by variability rather than a clear over- or underestimation (Thoenes and Oberfeld, 2017), can be affected by attention or working memory impairments (e.g., Campbell and Davalos, 2015), and hence are difficult to interpret at an individual level (see Giersch and Mishara, 2017b, for a discussion on implicit vs. explicit measures in schizophrenia).

The temporal expectation task explores the ability to continuously extract temporally predictive information from the elapse of time, i.e., to benefit from the predictive information inherent in the unidirectional flow of time. A disconnection from time's flow would mean a lack of immersion in the world, which should impact the patient's ability to feel present and embodied in his actions, feelings and thoughts. The observation of both types of impairment in this patient would reinforce the hypothesis of a link between implicit timing and the sense of self.

### AF case report

#### Anamnesis and symptomatology

AF is a 25-year-old man, who has never worked since his professional training in electronics. He attends a rehabilitation center because of social and professional difficulties. He has been followed psychiatrically for 4 years, following a suicide attempt and a diagnosis of schizophrenia. When we met him, he had no more psychiatric support and no treatment. A written informed consent was obtained from AF for the publication of this case report.

AF was born in Lyon (France). He was adopted at the age of 2 years. His childhood contained no great peculiarities, though he describes himself as “shy” since childhood. He has always been imbued with the diffuse feeling of “being different.” During childhood, he was a good pupil. The family atmosphere was serene. However, a state of psychic suffering gradually settled in during early adulthood. It was marked by a difficulty in maintaining good school performance (“everything required more and more effort…maybe I became too analytical”). AF also describes growing difficulty in interacting with others “I gradually became isolated…It is as if contact with others was not natural anymore…I began to wonder how others behaved so naturally.” AF adds: “Everything was a question for me…stupid questions like ‘why does the mouth go upwards when you smile’.” He gradually quit school, became isolated and withdrew to the family home. At the age of 18, he attempted suicide “As amazing as it may seem…I could no longer stand to always be thinking about everything.” He was then hospitalized for 3 months in a psychiatric ward for a delirious state, marked by ideas of persecution. Antipsychotic drugs (risperidone and then olanzapine) were then prescribed and the diagnosis of schizophrenic disorder was made on the presence of delusions, negative symptoms, major impairments in interpersonal functioning, and lack of evidence for schizoaffective disorder and autism.

When we encountered AF, functional impairments persisted, i.e., difficulty in social and professional integration, but there was no longer any obvious behavioral symptomatology (see clinical details in Supplementary Material). The patient voiced two major complaints. The first concerned the feeling of being oneself, and the second his experience of time.

#### Lack of ownership—minimal self disorder (SDs)

On several occasions, AF reported feelings of being at a distance from his environment and his own sensation of being a subject.

“I often observe myself from the outside…When I'm speaking, I'm seeing myself speaking at the same time that I'm speaking…when I'm walking, it is as if I am controlling myself artificially at the same time that I am walking…as if I were outside myself, and it is not really I who perceive…as if my perception is bland, artificial…as if I am not there….”

In addition, AF described a loss of natural evidence, associated with an experience of hyper-reflexivity. “Everything raises questions…it is as if the world would be so far from me that everything is questionable…why is the table called a table…why is the sky blue…why do I have two arms and not three.”

Finally, AF described: “it is as if the content of my perception is wrong…as if there is me on one side, and an artificial environment on the other side that reflects my perception…as if what I perceive is not really what is there to be perceived.…I sometimes have the impression that it is cut in two parts…or that I'm alone, without a real perception.”

AF's bodily feelings, and relationships to his own thoughts were also disturbed, and descriptions can be found in Supplementary Materials.

#### Disturbance of temporality

Timing disturbances are usually difficult to explore, but come up frequently in AF's complaints: “I do not feel the time,” “The word ‘time’ has no meaning for me…[I have to] use tools, tricks, to know that time has passed.” “For example, I often look at my watch to know that time elapsed…” “You see, I can use a metaphor to explain to you…Birds, they have a sense that allows them to orient themselves…a kind of magnetism…It is an innate thing…If they do not have it, they cannot navigate…Me, it's the time I do not have…I'm like blind to time…but I cannot explain it better…I try to find out how to talk about it…but I can't manage to explain… It may be the most important thing to understand…”

Although time is his main complaint, AF is unable to better specify the particularity of his relationship to time.

## Other investigations—materials and methods

### EASE scale

The EASE is a 57 item semi-structured interview designed to explore SDs. The scale was administered by BM, who was trained in its use by one of the authors of the EASE scale (JP). Five domains are explored, as detailed in Supplementary Materials.

AF had a total score of 16, which corresponds to a score frequently found in the population of people with schizophrenia. Detailed sub-scores can be found in Supplementary Material.

### Neuro cognition

A neurocognitive assessment was performed by a neuropsychologist, including psychomotor speed, working memory, verbal memory and executive functions. No major deficit was observed, except a flexibility deficit. Scores are detailed in Table 1.

**Table 1:** Scores of AF in the neurocognitive battery.

**ATTENTIONAL FUNCTIONING (D2) (Brickenkamp, 2002)**	
KL (concentration)	184 (RP 57,9)
GZ (speed)	445 (RP 30,9)
**FLEXIBILITY (TMT) (Tombaugh, 2004)**	
TMT A (speed)	32 (DS−0,9)
TMT B (flexibility)	96 (DS = −3,7)^*^
TMT A – B	64 (DS = −1,7)^*^
Short term memory (forward digit span – WAIS 4)	7 (DS = +0,4)
Working memory (backward digit span – WAIS 4)	6 (DS = +1)
**VERBAL LONG TERM MEMORY (FREE AN CUED SELECTIVE REMINDING TEST—RL/RI-16) (Grober and Buschke, 1987)**	
Immediate recall	16 (RP 50)
Free recall 1	14 (DS +1,5)
Free recall 2	14 (DS = +1,5)
Free recall 3	16 (DS +0,7)
Delayed Free recall	15 (DS +0,16)
Total recall 1	16 (RP 75)
Total recall 2	16 (RP 50)
Total recall 3	16 (RP 25)
Total delayed recal	16 (RP 25)
**MULTIPLE ERRANDS TEST (MET) (Shallice and Burgess, 1991)**	
Total number of errors	2 (RP 50)
Verbal reasoning	27 (NS = 14)

*The battery was designed to explore the main cognitive functions known to be impaired in patients with schizophrenia (Heinrichs and Zakzanis, 1998), and known to affect explicit duration estimation in these patients (Bonnot et al., 2011; Roy et al., 2012; Ward et al., 2012; Campbell and Davalos, 2015; de Montalembert et al., 2016). It is to be noted that the patient did not display thymic disorders (see Supplementary Material for details)*.

### Temporal prediction task

A visual target was displayed at various intervals after an initial fixation point (400 or 1,000 ms), and the task was to press a response key as quickly as possible once the target was displayed. The conditional probability of target presentation increases with the length of the interval (termed the “hazard function”), resulting in a heightening sense of expectation over time. Increasing expectation results in shorter reaction times (RTs) as the delay between fixation point and target increases (Niemi and Näätänen, 1981). This decrease in RT is referred to as the variable foreperiod effect.

To investigate the patient's ability to implicitly take such conditional probabilities into account, he performed the task in two different types of experimental blocks. In one set of blocks, the initial fixation point was not followed by the target in 25% of trials (25% catch trials) whereas in the other blocks the target always followed the fixation point (0% catch trials). Catch trials decrease the expectancy that the target will be presented and lead to “dispreparation,” yielding a decrease in the variable foreperiod effect.

**Figure 1:**
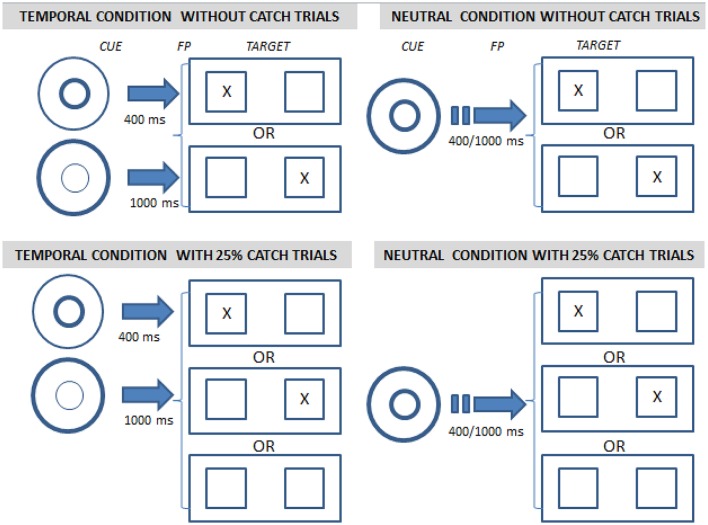
Illustration of the conditions used in the temporal orienting task. After the initial central fixation point (on the left in each quadrant), the target was displayed left or right of screen center (on the right in each quadrant) and subjects had to press a response key corresponding to the side of target presentation as quickly and accurately as possible. The fixation point comprised a cue indicating the delay between fixation and target (400 or 1,000 ms, “temporal condition,” two left quadrants) or not (“neutral condition,” two right quadrants). In addition the target was either always displayed after the fixation point (0% catch trials, 2 upper quadrants) or was absent in 25% of the cases (25% catch trials, 2 lower quadrants).

We also investigated the ability to voluntarily orient attention in time. In half of the blocks, we used visual cues to indicate the time of occurrence of the target, either 400 or 1,000 ms after the fixation point (Figure 1). This temporal cue condition was contrasted with a neutral cue condition (which provided no temporal information).

All experimental conditions were crossed orthogonally (Figure 1). A detailed description is provided in Supplementary Materials.

To further explore the implicit processing of time intervals we analyzed trial-to-trial sequential effects in blocks with 0% catch trials (Los and van den Heuvel, 2001). These effects rely on automatic mechanisms (Vallesi et al., 2014), whereby the time interval of the preceding trial influences performance on the current trial: if the foreperiod on trial N is shorter than that on trial N-1, reaction times are slowed. However, no such effect is observed when the foreperiod is longer on trial N than N-1.

The temporal orienting test has been used widely (Coull and Nobre, 1998; Correa et al., 2006; Nobre and van Ede, 2018) and participants matched to AF on age and education level showed typical results on this test: RTs were faster for temporal vs. neutral cue conditions and, in the neutral condition, for long vs. short foreperiods (Martin et al., 2017).

## Results—temporal prediction task

**Figure 2:**
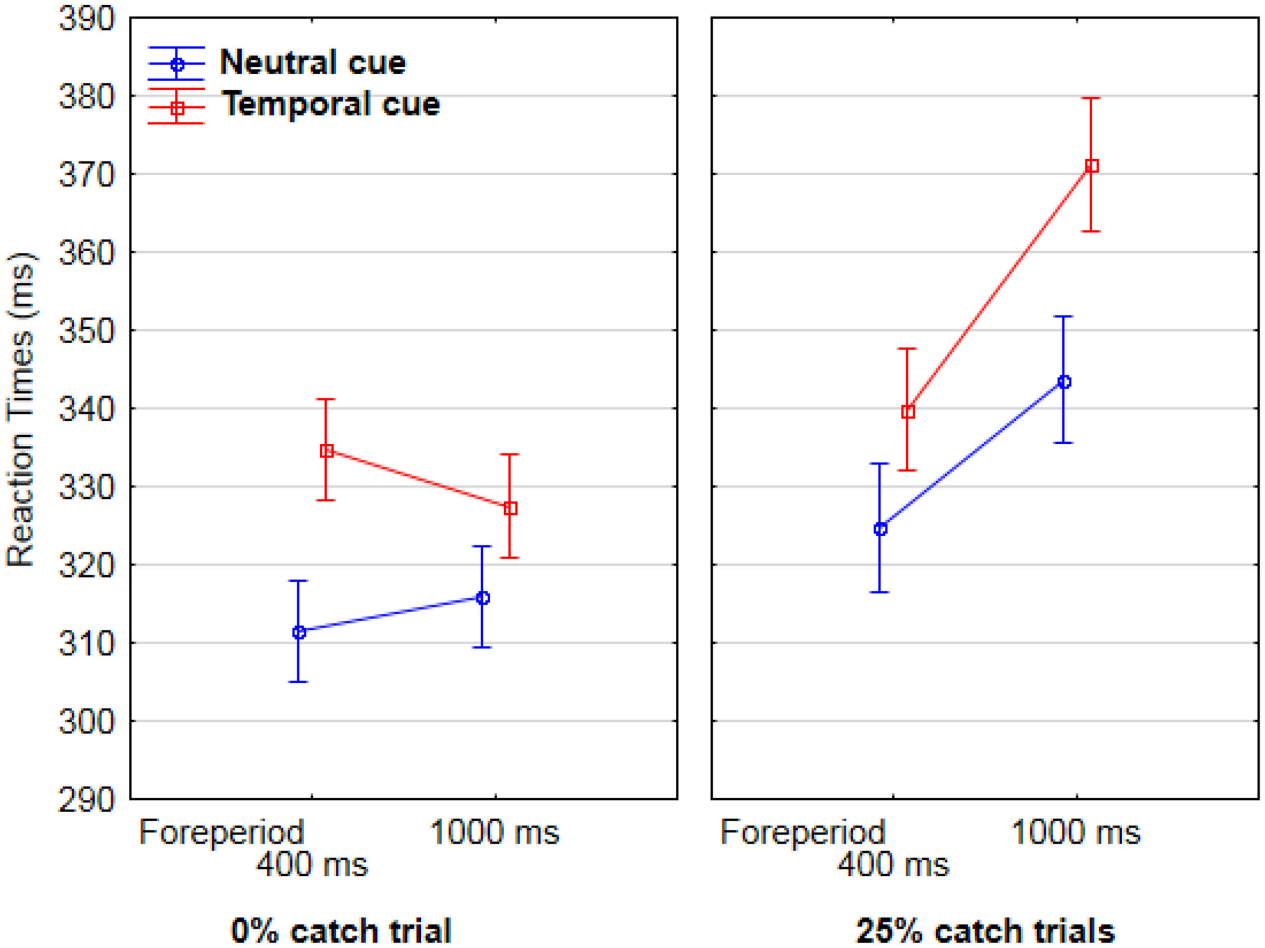
Mean Reaction times for AF as a function of catch-trial condition (0% catch trials in the lefthand graph, vs. 25% catch trials in the righthand graph), foreperiod between cue and target (400 vs. 1,000 ms, on the X axis), and cue type (temporal in red and neutral in blue).

AF committed only 3.3% errors, which were not analyzed further. Analyses of variance were conducted on RTs for correct trials, with each RT as a random variable. In the first analysis we included cue type (temporal vs. neutral), catch-trial percentage (0% vs. 25%), and foreperiod (400 vs. 1,000 ms) as between-group variables. Results showed a significant interaction between foreperiod and catch-trial percentage [*F*_(1, 752)_ = 6.5, *p* < 0.05). *Post-hoc* Tukey analysis showed that in case of 0% catch trials there was no difference in RTs between 400 ms (323 ms) and 1,000 ms (321 ms), whereas in the case of 25% catch trials, RTs increased between 400 ms (333 ms) and 1,000 ms (357 ms), p <.05. There was also a main effect of cue type [*F*_(1, 752)_ = 13.8, *p* < 0.001]. RTs were longer for temporal (340 ms) than for neutral cues (321 ms) (Figure 2).

**Figure 3:**
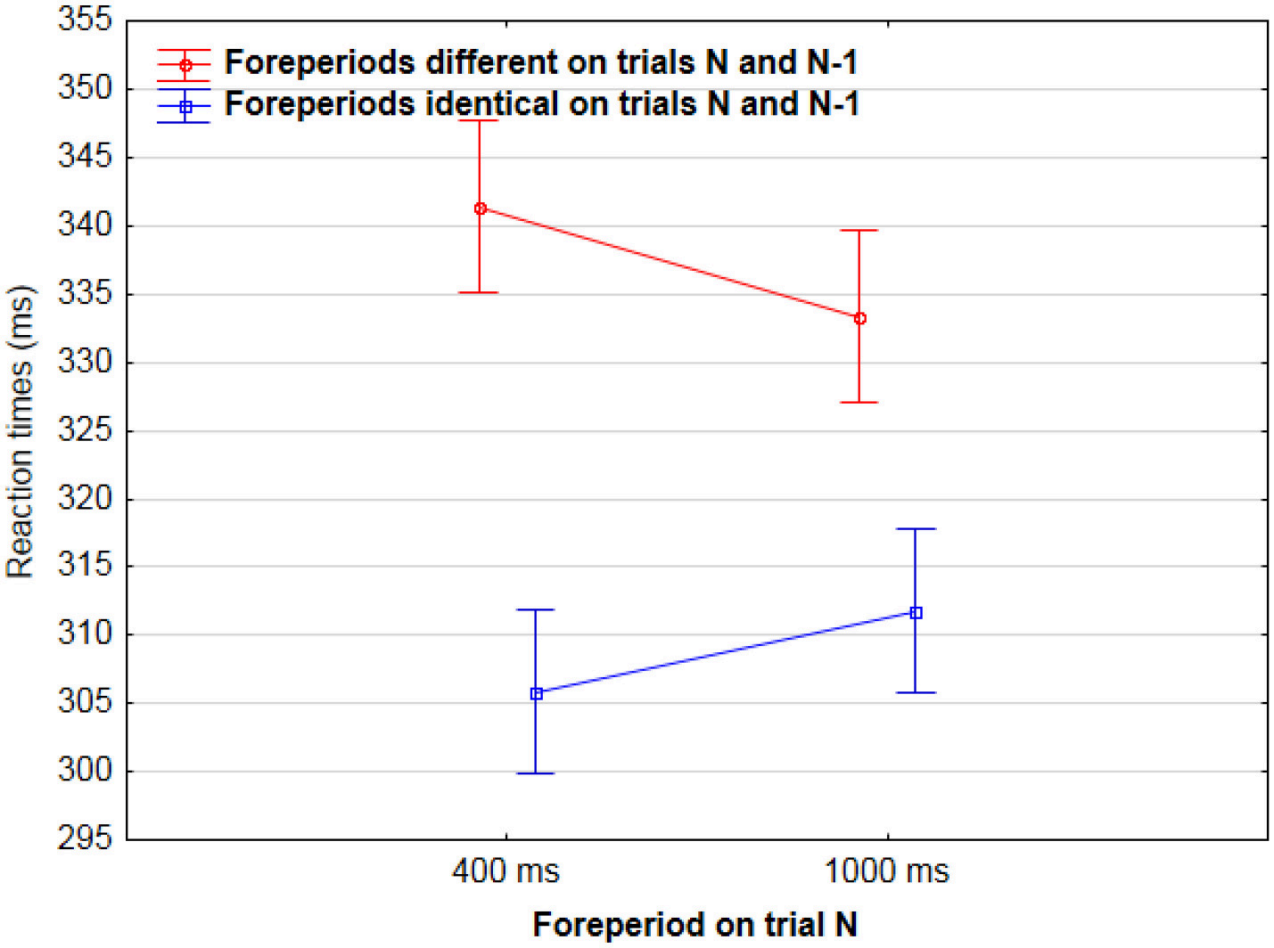
Mean Reaction times for AF on trial N as a function of foreperiod duration on trial N (400/1,000 ms) and of sequence type: foreperiods were either different (red) or identical (blue) in trials N and N-1.

In a second analysis, we analyzed sequential effects during neutral cue 0% catch trial blocks only. Results showed that RTs were longer when the foreperiod of two consecutive trials were different rather than identical [337 vs. 307 ms, *F*_(1, 444)_ = 21.4, *p* < 0.001]. This effect was significant both when the foreperiod was 400 ms on trial N [342 vs. 305 ms, *F*_(1, 224)_ = 18.9, *p* < 0.001], and when it was 1,000 ms [333 vs. 311 ms, *F*_(1, 221)_ = 5.4, *p* < 0.05] (Figure 3).

## Discussion

The case of AF is characterized by low intensity schizophrenia symptoms. In particular, AF shows no delusion or disorganization of thought. However, AF presents significant subjective complaints that can be attributed to SDs. A normal sense of self involves automatic, not-reflexive, self presence, and immersion in the world (Parnas et al., 2005). We perceive and act in the world “from the inside.” Because our thinking is “glued” to ourselves, our consciousness is non-spatial in nature and we cannot locate our consciousness, our thoughts, which are intuitively experienced as ours.

In contrast, AF describes experiences reflecting a distance from his own perception and self-awareness, i.e., a lack of immersion in the world. His own consciousness of himself, as well as his own perceptions, no longer seem embodied by the self and appear to him as mechanical and distinct from himself. In addition, the patient presents a loss of natural evidence, and a loss of immersion in his environment (Parnas et al., 2005). Finally, AF's descriptions of a “perceptualization of inner discourse” and “spatialization of thought” (see Supplementary Material) suggest that there is a gap between his thoughts and the self, reflecting and/or leading to the loss of the “mineness” of mental experience.

Taken together, these disturbances reflect a basic alteration of the feeling of presence, i.e., immersion in the world, which represents a fundamental feature of SDs. AF's disturbances are attested by his high score on the EASE scale.

AF had a normal neuro-cognitive assessment, despite his minimal self disturbances being quite large. This dissociation between normal neurocognitive outcome and SDs is consistent with the literature (Haug et al., 2012b), which does not indicate a correlation between typical neuro-cognitive disturbances and minimal SDs.

Usual neuro-cognitive batteries are not exhaustive though, and AF insists that he does not “have a sense of time.” AF also insists on his great difficulty in being able to describe more precisely the peculiarities of his time experience.

### AF shows several timing impairments

AF does not benefit from the flow of time to optimize his temporal expectancies, i.e., his reaction time does not decrease as the probability of target occurrence increases. In addition, in the presence of catch trials, there is a large dispreparation effect (Correa et al., 2006) resulting in slower RTs at 1,000 ms than at 400 ms. These results are consistent with those observed at the group level (Martin et al., 2017), especially in patients with SDs. As in this previous study, our results suggest a fragility in the ability to make temporal predictions. Moreover, results fit with AF's complaint that he does not feel that time has elapsed. Despite these difficulties, some basic ability to encode time intervals is preserved, as shown by the fact that, as in healthy participants, RTs increase when the foreperiod between consecutive trials differs. This result shows that the difference between 400 and 1,000 ms has been encoded. This is not surprising, since automatic coding of short durations has been found in electrophysiological studies of cortical slices, i.e., without requiring the entire cortex (Goel and Buonomano, 2016). In AF, it is not interval estimation that is impaired, but the ability to use this interval to predict the probability of target occurrence. It is also to be noted that AF had no difficulty in taking probabilities into account, since performance changed when the probability of target presentation was manipulated, i.e., in the case of catch trials. It is thus the temporal prediction itself that seems affected.

However, some of AF's results had not been observed at the group level. Sequential effects were preserved in our group of patients with schizophrenia (see Supplementary Material). For AF, performance was similarly affected whether the foreperiod on trial N was shorter *or longer* than that on trial N-1. It's as if AF expected the foreperiod to be strictly identical on consecutive trials and was unable to reinitialize expectation once the target had not been displayed after the short foreperiod. This effect is reminiscent of previous studies suggesting heightened sequential effects in patients (Zahn et al., 1963). Together with AF's difficulty in using temporal cues (unusually, RTs were slower, rather than faster, after temporal cues), the results suggest that temporal prediction is especially fragile and lacks flexibility in this patient.

Overall, AF's complaints about time were supported by objective exploration. The paucity of other symptoms and neuro-cognitive impairment makes it tempting to propose a relationship between objective and/or subjective timing difficulties and minimal SDs, consistent with previous hypotheses (Martin et al., 2014, 2017; Giersch and Mishara, 2017a,b). However, such a relationship cannot be proven, and more extensive exploration of timing might be useful, e.g. using longer time intervals. Yet, the important result in the present study is that timing disorders can be demonstrated at an individual level, independent of antipsychotics or impairments in attention and memory. This is the first step toward personalized evaluation and therapeutics. We suggest that subjective and objective explorations of timing may usefully complete patients' clinical evaluation.

## Ethics statement

The project was approved by a local ethics committee (CPP Sud Est VI), and informed written consent was obtained, before the study, from each patient and control participant. All methods have been conducted in accordance with the recommendation of the Declaration of Helsinki.

## Author contributions

AG and JC: designed the study; BM and NF: provided their clinical expertise and acquired the experimental and clinical data; BM: conducted the clinical interviews; BM and MC: scored the EASE; AG: analyzed the experimental data; AG, JC, and BM: interpreted the data; BM: wrote the first draft of the manuscript; AG and JC: edited and finalized the manuscript. All authors reviewed, amended, and approved the manuscript.

### Conflict of interest statement

The authors declare that the research was conducted in the absence of any commercial or financial relationships that could be construed as a potential conflict of interest.

## References

[B1] Bonnot O. de Montalembert M. Kermarrec S. Botbol M. Walter M. Coulon N. (2011). Are impairments of time perception in schizophrenia a neglected phenomenon? J. Physiol. Paris 105, 164–169. 10.1016/j.jphysparis.2011.07.006 21803155

[B2] Brickenkamp R. (2002). Test d2. Aufmerksamkeits-Belastungs-Test (9th Edn). The d2 test. Test of Attention Under Pressure. Göttingen: Hogrefe.

[B3] Campbell A. M. Davalos D. B. (2015). Levels of attention and task difficulty in the modulation of interval duration mismatch negativity. Front. Psychol. 6:1619. 10.3389/fpsyg.2015.01619 26579010 PMC4621395

[B4] Correa A. Lupiáñez J. Tudela P. (2006). The attentional mechanism of temporal orienting: determinants and attributes. Exp. Brain Res. 169, 58-68. 10.1007/s00221-005-0131-x 16273403

[B5] Coull J. T. Nobre A. C. (1998). Where and when to pay attention: the neural systems for directing attention to spatial locations and to time intervals as revealed by both PET and fMRI. J. Neurosci. 18, 7426–7435. 9736662 10.1523/JNEUROSCI.18-18-07426.1998 PMC6793260

[B6] Coull J. T. Nobre K. (2008). Dissociating explicit timing from temporal expectation with fMRI. Curr. Opin. Neurobiol. 18, 137–144. 10.1016/j.conb.2008.07.011 18692573

[B7] de Montalembert M. Coulon N. Cohen D. Bonnot O. Tordjman S. (2016). Time perception of simultaneous and sequential events in early-onset schizophrenia. Neurocase 22, 392–399. 10.1080/13554794.2016.1205098 27388526

[B8] Fuchs T. (2007). The temporal structure of intentionality and its disturbance in schizophrenia. Psychopathology 40, 229-235. 10.1159/000101365 17396049

[B9] Gallagher S. (2000). Philosophical conceptions of the self: implications for cognitive science. Trends Cogn. Sci. 4, 14-21. 10.1016/S1364-6613(99)01417-5 10637618

[B10] Gallese V. Ferri F. (2013). Jaspers, the body, and schizophrenia: the bodily self. Psychopathology 46, 330-336. 10.1159/000353258 23867974

[B11] Giersch A. Mishara A. L. (2017a). Disrupted continuity of subjective time in the milliseconds range in the self-disturbances of schizophrenia: convergence of experimental, phenomenological and predictive coding accounts. J. Consc. Studies 24, 62–87.

[B12] Giersch A. Mishara A. L. (2017b). Is schizophrenia a disorder of consciousness? experimental and phenomenological support for anomalous unconscious processing. Front. Psychol. 8:1659. 10.3389/fpsyg.2017.01659 29033868 PMC5625017

[B13] Goel A. Buonomano D. V. (2016). Temporal interval learning in cortical cultures is encoded in intrinsic network dynamics. Neuron 91, 320–327. 10.1016/j.neuron.2016.05.042 27346530 PMC4969202

[B14] Grober E. Buschke H. (1987). Genuine memory deficits in dementia. Dev. Neuropsychol. 3, 13–36. 10.1080/87565648709540361

[B15] Haug E. Lien L. Raballo A. Bratlien U. Oie M. Andreassen O. A.. (2012a). Selective aggregation of selfdisorders in first-treatment DSM-IV schizophrenia spectrum disorders. J. Nerv. Ment. Dis. 200, 632–636. 10.1097/NMD.0b013e31825bfd6f 22759943

[B16] Haug E. Øie M. Melle I. Andreassen O. A. Raballo A. Bratlien U.. (2012b). The association between self-disorders and neurocognitive dysfunction in schizophrenia. Schizophr. Res. 135, 79-83. 10.1016/j.schres.2011.11.015 22137461

[B17] Heinrichs R. W. Zakzanis K. K. (1998). Neurocognitive deficit in schizophrenia: a quantitative review of the evidence. Neuropsychology 12, 426–445. 9673998 10.1037//0894-4105.12.3.426

[B18] Hur J.-W. Kwon J. S. Lee T. Y. Park S. (2014). The crisis of minimal self-awareness in schizophrenia: a meta-analytic review. Schizophr. Res. 152, 58-64. 10.1016/j.schres.2013.08.042 24055201

[B19] Los S. A. van den Heuvel C. E. (2001). Intentional and unintentional contributions to nonspecific preparation during reaction time foreperiods. J. Exp. Psychol. Hum. Percept. Perform. 27, 370–386. 10.1037/0096-1523.27.2.370 11318053

[B20] Lysaker P. H. Lysaker J. T. (2010). Schizophrenia and alterations in self-experience: a comparison of 6 perspectives. Schizophr. Bull. 36, 331-40. 10.1093/schbul/sbn077 18635676 PMC2833111

[B21] Martin B. Wittmann M. Franck N. Cermolacce M. Berna F. Giersch A. (2014). Temporal structure of consciousness and minimal self in schizophrenia. Front. Psychol. 5:1175. 10.3389/fpsyg.2014.01175 25400597 PMC4212287

[B22] Martin B. Franck N. Cermolacce M. Falco A. Benair A. Etienne E.. (2017). Fragile temporal prediction in patients with schizophrenia is related to minimal self disorders. Sci. Rep. 7:8278. 10.1038/s41598-017-07987-y 28811493 PMC5557764

[B23] Mishara A. L. Lysaker P. H. Schwartz M. A. (2014). Self-disturbances in schizophrenia: history, phenomenology, and relevant findings from research on metacognition. Schizophr. Bull. 40, 5–12. 10.1093/schbul/sbt169 24319117 PMC3885311

[B24] Møller P. Husby R. (2000). The initial prodrome in schizophrenia: searching for naturalistic core dimensions of experience and behavior. Schizophr. Bull. 26, 217–232. 10755683 10.1093/oxfordjournals.schbul.a033442

[B25] Nelson B. Thompson A. Chanen A. M. Amminger G. P. Yung A. R. (2013a). Is basic self-disturbance in ultra-high risk for psychosis (“prodromal”) patients associated with borderline personality pathology? Early Interv. Psychiatry 7, 306–310. 10.1111/eip.12011 23347769

[B26] Nelson B. Thompson A. Yung A. R. (2013b). Not all first-episode psychosis is the same: preliminary evidence of greater basic self-disturbance in schizophrenia spectrum cases. Early Interv. Psychiatry 7, 200-204. 10.1111/j.1751-7893.2012.00381.x 22759705

[B27] Niemi P. Näätänen R. (1981). Foreperiod and simple reaction time. Psychol. Bull. 89, 133-162.

[B28] Nobre A. C. van Ede F. (2018). Anticipated moments: temporal structure in attention. Nat. Rev. Neurosci. 19, 34–48. 10.1038/nrn.2017.141 29213134

[B29] Nordgaard J. Handest P. Vollmer-Larsen A. Sæbye D. Pedersen J. T. Parnas J. (2017). Temporal persistence of anomalous self-experience: a 5years follow-up. Schizophr. Res. 179, 36–40. 10.1016/j.schres.2016.10.001 27720316

[B30] Nordgaard J. Parnas J. (2014). Self-disorders and schizophrenia spectrum: a study of 100 first hospital admissions. Schizophr. Bull. 40, 1300–1307. 10.1093/schbul/sbt239 24476579 PMC4193705

[B31] Parnas J. Handest P. Saebye D. Jansson L. (2003). Anomalies of subjective experience in schizophrenia and psychotic bipolar illness. Acta Psychiatr. Scand. 108, 126–133. 12823169 10.1034/j.1600-0447.2003.00105.x

[B32] Parnas J. Møller P. Kircher T. Thalbitzer J. Jansson L. Handest P. (2005). EASE: Examination of Anomalous Self-Experience. Psychopathology 38, 236-258. 10.1159/000088441 16179811

[B33] Raballo A. Sæbye D. Parnas J. (2011). Looking at the schizophrenia spectrum through the prism of self-disorders: an empirical study. Schizophr. Bull. 37, 344-351. 10.1093/schbul/sbp056 19528205 PMC3044618

[B34] Roy M. Grondin S. Roy M. A. (2012). Time perception disorders are related to working memory impairments in schizophrenia. Psychiatry Res. 200, 159–166. 10.1016/j.psychres.2012.06.008 22862910

[B35] Shallice T. Burgess P. W. (1991). Deficits in strategy application following frontal lobe damage in man. Brain 114, 727–741. 10.1093/brain/114.2.727 2043945

[B36] Stanghellini G. (2009). Embodiment and schizophrenia. World Psychiatry 8, 56-59. 19293962 10.1002/j.2051-5545.2009.tb00212.x PMC2652898

[B37] Thoenes S. Oberfeld D. (2017). Meta-analysis of time perception and temporal processing in schizophrenia: differential effects on precision and accuracy. Clin. Psychol. Rev. 54, 44–64. 10.1016/j.cpr.2017.03.007 28391027

[B38] Tombaugh T. N. (2004). Trail making test A and B: normative data stratified by age and education. Arch. Clin. Neuropsychol. 19, 203–214. 10.1016/S0887-6177(03)00039-8 15010086

[B39] Vallesi A. Arbula S. Bernardis P. (2014). Functional dissociations in temporal preparation: evidence from dual task performance. Cognition 130, 141–151. 10.1016/j.cognition.2013.10.006 24291265

[B40] Vogeley K. Kupke C. (2007). Disturbances of time consciousness from a phenomenological and a neuroscientific perspective. Schizophr. Bull. 33, 157–165. 10.1093/schbul/sbl056 17105967 PMC2632289

[B41] Ward R. D. Kellendonk C. Kandel E. R. Balsam P. D. (2012). Timing as a window on cognition in schizophrenia. Neuropharmacology 62, 1175–1181. 10.1016/j.neuropharm.2011.04.014 21530549 PMC3155658

[B42] Zahn T. P. Rosenthal D. Shakow D. (1963). Effects of irregular preparatory intervals on reaction time in schizophrenia. J. Abnorm. Soc. Psychol. 67, 44–52. 10.1037/h0049269 14003031

