# Supplementary material for: Minimal Self and Timing Disorders in Schizophrenia: A Case Report
Source: Front Hum Neurosci. 2018 Apr 6;12:132. doi: 10.3389/fnhum.2018.00132 (PMC5900747; doi:10.3389/fnhum.2018.00132)
Supplement: Supplementary file 1 [file DataSheet1.DOCX]

**Supplementary material**

**MATERIAL and METHODS**

**Participants**

The project was approved by a local ethics committee (CPP Sud Est VI), and informed written consent was obtained, before the study, from each patient and control participant. All methods have been conducted in accordance with the recommendation of the Declaration of Helsinki.

Psychiatric diagnoses were established by a senior psychiatrist from the department. Diagnoses fulfilled the Diagnostic and Statistical Manual of Mental Disorders, Fifth Edition, critera for a diagnosis of schizophrenia. Exclusion criteria for patients and controls were the intake of benzodiazepines, a history of alcohol and drug dependency, neurological and medical pathologies, a disabling sensory disorder, and general anesthesia in the 3 months prior to testing. An additional exclusion criterion for controls was psychotropic medication in the 3 weeks prior to testing.

**Ease Scale**

The 5 domains explored in the EASE scale are the following:

- Cognition and stream of consciousness (17 items). This domain explores disturbances in the stream of consciousness associated with the feeling of a gap between one’s own thoughts and the self, leading to the loss of “mineness” of mental experience.
- Self awareness and presence (18 items). This domain explores a broad range of phenomena that can be defined as a lack of immersion in the world.
- Bodily experiences (9 items). This domain explores a broad range of bodily experiences characterized by the feeling of being detached from oneself and one’s actions, as if in a third- person perspective or without any perspective at all.
- Demarcation – transitivism (5 items). This domain explores a range of experiences characterized by a difficulty discriminating self from not self
- Existential reorientation (8 items). The patient manifests a fundamental reorientation with respect to his general metaphysical world view and/or hierarchy of values, projects and interests.

Each item is scored on a 2 point likert scale: 0 for absent or questionably present and 1 for definitely present. The interview took 120 min, and was video filmed

**Temporal orienting task**

The paradigm has been detailed in Martin et al. (2017). It should be noted that the control group in Martin et al. (2017) matches AF globally on age and education level, and we present only controls’ results that had not been published in the original paper.

***Apparatus and stimuli***

The experiments were generated with E Prime2.0 on a Pentium 4 PC, and displayed on a 60 Hz monitor, with a low-intensity ambient light (0.1 cd/ m^2^).

A fixation point was constantly displayed in the centre of the screen, surrounded by two circles. Two empty squares were also permanently displayed left and right of centre (Figure 1). At the beginning of the trial, one or both of the two circles surrounding the fixation point was highlighted for 100 ms. After a delay of either 400 or 1000 ms (foreperiod), the target was shown in the left or right box for 100 ms. Participants were required to press on a response key as quickly as possible after target presentation and within a maximal interval of 2000 ms.

The highlighting of only the inner or outer circle represented the temporal cue and indicated that the target would appear after a short (400ms) or long (1000ms) foreperiod, respectively. The highlighting of both circles represented the neutral cue, which indicated that the target could appear after either 400 or 1000 ms.

***Procedure***

Participants were instructed to press a key corresponding to the side of the target presentation, as quickly and accurately as possible. They started with 64 practice trials, allowing them to learn the association between the temporal cues and foreperiod duration (400 or 1000 ms). Training was followed by eight blocks of 120 experimental trials.

Four blocks contained 0% catch trials, and four contained 25% catch trials. In the four blocks without catch trials (0% catch trial), there were two blocks with temporal cues (T) and two with neutral cues (N). The same procedure was applied to the four blocks with catch trials (25% catch trials). AF performed the experiment in the order TNNT. The order of blocks with, or without, catch trials was randomized across control participants, as was the order of temporal versus neutral cue blocks.

***Statistical analysis***

Correct responses faster than 150 ms or slower that 1000 ms were excluded from the analyses (<3% trials).

In the group analysis, RTs were averaged for each condition and each subject, and mean RTs were random variables in the analyses of variance (ANOVA). Foreperiod (400ms/1000ms), cue type (temporal/neutral) and catch-trial percentage (0%/25%) were within-subjects variables, and the group (patients vs. controls) was the between-subjects variable. In the single-subject analysis, RTs were the random variables in the analyses of variance (ANOVA), while foreperiod (400ms/1000ms), cue type (temporal/neutral) and catch-trial percentage (0%/25%) were between-subjects variables. We decomposed interactions by means of Tukey post hoc analyses.

**Simultaneity-asynchrony discrimination task**

***Subjects***

In addition to AF, 10 controls with a mean age and education level close to those of AF were included to evaluate asynchrony detection.

Eight of the controls matched to AF had been part of the control group in Martin et al. (2017) and two have been added. The mean age of the group of 10 controls was 23,8 years (SD 3.8) and the level of education was 13.8 years (SD 1.4).

***Equipment and stimuli***

We explored the ability to discriminate whether two visual stimuli are simultaneous or asynchronous.

Two rectangles were displayed vertically (0.5 x 1.5°), 4.5° right and left from a central fixation point. Stimuli were generated on a CRT 60 Hz screen with a Pentium PC 4 programmed with Matlab 7.0.1 (Mathworks, 1984–2004) and psychophysics Toolbox extensions (Brainard, 1997; Pelli, 1997). Upon presentation, the rectangle luminance increased from 0.03 (background luminance) to 12 cd/m², over a presentation interval of 119 ms. The gradual increase in luminance was intended to avoid magno-cellular pathway activation (Giersch et al., 2009; Capa et al., 2014). The rectangles were displayed with a stimulus onset asynchrony (SOA) varying between 0 and 133 ms, by steps of 16.7 ms. Left-right and right left presentation were equally represented, and there was 40 trials for each SOA.

***Statistical analyses***

Thresholds were calculated as in previous studies (Giersch et al., 2009; Lalanne et al., 2012a,b), and represent the SOA corresponding to a rate of 50% simultaneous responses. To take into account false alarms, data was corrected by dividing all rates of simultaneous responses by the highest rate of synchronous responses in each subject (see Giersch et al., 2009 for details). The following analyses were all performed on corrected data.

***Evaluation of the Simon effect***

The measure of implicit timing during the simultaneity/asynchrony task is based on the Simon effect, which refers to the fact that subjects tend to respond to the side of stimulus presentation even if the task does not require such spatial information to be processed. This effect depends upon spatial asymmetry, which is not necessarily present when stimuli are presented simultaneously on both sides of the screen. In the case of two stimuli displayed simultaneously on the right and left side, no Simon effect can occur. However, when stimuli are asynchronous, there is once more an asymmetry between left and right, due to the temporal delay. As a matter of fact, Lalanne et al. (2012a,b) have shown a bias to respond to the side of the second stimulus in healthy volunteers (Lalanne et al., 2012a,b). More recent studies suggest that healthy subjects can follow stimuli in time at a non-conscious level, i.e. even when asynchronies are sub-threshold (Poncelet et al., 2015). A Simon effect was also present in patients with schizophrenia (Lalanne et al., 2012a, b). However, for sub-threshold asynchronies, the responses of the patients were biased to the side of the first stimulus, and not the second one. It is as if at an implicit level patients process stimuli as if they are isolated rather than in succession. This impairment has been proposed to be related to disturbed predictive coding. When stimuli are less than 20 ms apart, time is too short to shift attention to the second stimulus once the first has been displayed. Processing this sequence of stimuli efficiently requires that the system is prepared to process a second stimulus in advance. This might be based on the anticipation of a sequence of stimuli (Giersch et al., 2016). The Simon effect to the side of the 1^st^ stimulus may thus signal impairment of this anticipation mechanism in patients.

**RESULTS**

**AF clinical details**

| **Hospitalizations (duration)** | **Previous treatment** | **Current treatment** |
| --- | --- | --- |
| 2006 (3 months) | Antipsychotic drugs (risperidone, olanzapine) | No medication (refuses medication since 4 years) |
| 2009 (3 months) | Social skill training |  |

**Table S1:** Hospitalization and treatment history of AF

| **EASE sub-scores** | |  |
| --- | --- | --- |
| Cognition and stream of consciousness | | 4 |
| Self awareness and presence | | 9 |
| Bodily experiences | | 1 |
| Demarcation – transitivism | | 1 |
| Existential reorientation | | 1 |
| Total | | 16 |
| **PANSS sub-scores** |  |  |
| Positive | | 12 |
| Negative | | 16 |
| Global | | 27 |
| Total | | 55 |

**Table S2:** AF’s clinical scores

**AF’s relationship to his own thoughts – Perceptualization and spatialization of Inner Speech**

AF describes various experiences characterized by the feeling of a distance between himself and his thoughts. "I have the impression that my thought stream is like a sound ... It always accompanies me, in a sonorous way ... a bit like a symbiotic entity ". He insists, however, on the fact that he does not hear voices and does not have delusions.
AF locates his thoughts: "When I think, I feel very precisely where it is going ... it is all in the front of my head ... there, you see ..." (he shows his forehead). "I imagine that there must be brain circuits here ... it is in these circuits that the thought unfolds". “Besides, I feel that my thoughts, once born, take their source in the back of the skull and come to the front."

**AF Relationship to his own body**

AF describes various experiences during which he feels that his own bodily experience escapes him. In particular he perceives the functioning of his own organs. He says things like "feeling the blood flowing in my veins" or "my neurons moving in my brain when I think". These sensations lead him to express the following impression: "there is a whole world to which I do not belong, which moves within me".

Self-awareness is never a purely cognitive phenomenon but is embodied through the "lived body" (the "Leib" in German, as opposed to "Körper"). In this case, the experience of the body takes place, so to speak, "from the inside", in an "immanent" and "in a silent" way. For Stanghellini, in a perspective close to Merleau-Ponty, the experience of the "lived body" is consubstantial of the minimal self-awareness. "To be conscious," says Merleau-Ponty (1945), "is to be to the world through the lived body". AF describes a predominance of experiencing the body as a physical object. This type of experience, which can be called “spatialization of bodily experience”, reflects an alteration of the Leib, that is to say, the experience of embodiment.

**Results and discussion of the Simultaneity/Asynchrony task**

The threshold for asynchrony detection for AF was 46 ms. It was smaller than the threshold observed in the 10 age and education level-matched controls (60 ms, SE 1,6).

The Simon effect was analyzed for the two shortest sub-threshold SOAs, i.e. 17 and 33 ms. There was a Simon effect to the side of the 1^st^ stimulus in AF: simultaneous responses were more frequent in 25% of cases in the left-right than in the right-left direction (Chi² =5.95, df=1, p<.05). There was no significant Simon effect in controls (4% more simultaneous responses in the left-right than the right-left direction, F(1,8)=3.5, n.s.). The amplitude of the Simon effect in patient AF is significantly larger than in controls (Chi² =6.27, df=1, p<.05).

AF displayed a good ability to discriminate asynchronies but a large Simon effect to the side of the first stimulus. His results are consistent with the difficulties in anticipating sequences of stimuli described in several groups of patients in previous studies (Lalanne et al., 2012a,b). These difficulties are consistent with his time prediction impairment.

**Complementary results in the temporal orienting task: sequential effects**

Variable foreperiod, temporal orienting and catch trials effects have already been published in Martin et al (2017). We show here sequential effects in the group of control participants that had not been reported in the original paper, but have since been analyzed in AF.

As for AF, we analysed sequential effects in neutral cue 0% catch trials blocks. Averaged RTs were submitted to an ANOVA, with foreperiod on trial N (400 ms/1000 ms), cue type (temporal/neutral) and sequence type (identical vs. different foreperiods on trials N and N-1) as within-group variables, and group (controls vs. patients) as a between-group variable.

The analysis showed a significant interaction between foreperiod and sequence type (F[1, 49]=100, p<.001, without any interaction with group (F<1, Figure S1). Post-hoc Tukey analyses showed that RTs were longer when foreperiods were different rather than identical on trials N and N-1, but only when the foreperiod of trial N was 400 ms (and 1000 ms on trial N-1). This effect was significant both in controls and in patients (ps<.001 in both groups).


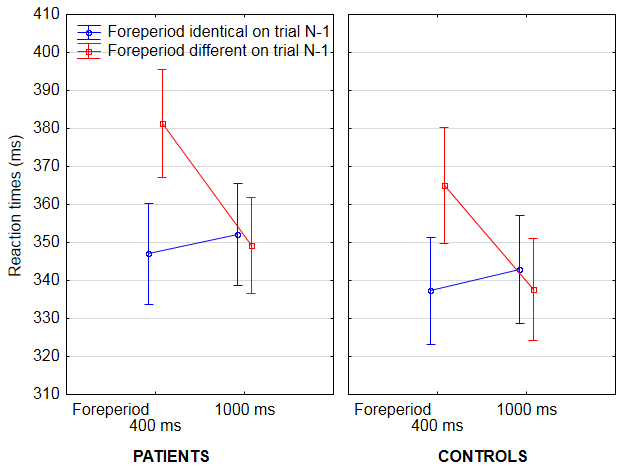


**Figure S1**: Reaction times in patients (lefthand graph) and controls (righthand graph) as a function of the foreperiod between the cue and the target (400 vs. 1000 ms), and the sequence type (foreperiods different –in red- vs. identical –in blue- in trials N and N-1).

Both patients and controls showed the typical sequential effects (ref), i.e. a lengthening of RTs when the target was displayed earlier in trial N than in trial N-1. Contrary to AF, there was no lengthening of RTs in the opposite condition, i.e. when the target was displayed later in trial N than in trial N-1.

**References**

Brainard, D.H. (1997). The Psychophysics Toolbox. *Spat. Vis.* 10, 443-446.

Capa, R.L., Duval, C.Z., Blaison, D., and Giersch, A. (2014). [Patients with schizophrenia selectively impaired in temporal order judgments.](http://www.ncbi.nlm.nih.gov/pubmed/24768441) *Schizophr. Res*. 156, 51-55. doi: 10.1016/j.schres.2014.04.001.

Giersch, A., Lalanne, L., Corves, C., Seubert, J., Shi, Z., Foucher, J., and Elliott, M.A. (2009). [Extended visual simultaneity thresholds in patients with schizophrenia.](http://www.ncbi.nlm.nih.gov/pubmed/18359954) *Schizophr. Bull.* 35(4), 816-825. doi: 10.1093/schbul/sbn016.

Lalanne, L., van Assche, M., and Giersch, A. (2012a). When predictive mechanisms go wrong: disordered visual synchrony thresholds in schizophrenia. *Schizophr. Bull.* 38(3), 506-513. doi: 10.1093/schbul/sbq107.

Lalanne, L., Van Assche, M., Wang, W., and Giersch, A. (2012b). Looking forward: an impaired ability in patients with schizophrenia? *Neuropsychologia* 50(12), 2736-2744. doi: 10.1016/j.neuropsychologia.2012.07.023.

Martin, B., Franck, N., Cermolacce, M., Falco, A., Benair, A., Etienne, E., et al. (2017). Fragile temporal prediction in patients with schizophrenia is related to minimal self disorders. *Sci. Rep.* 7, 8278, doi : 10.1038/s41598-017-07987-y.

Merleau-Ponty, M. (1945). Phénoménologie de la perception. Paris: Gallimard.

Pelli, D.G. (1997). The VideoToolbox software for visual psychophysics: Transforming numbers into movies, *Spat. Vis*. 10, 437-442.

Poncelet, P.E., and Giersch, A. (2015). [Tracking Visual Events in Time in the Absence of Time Perception: Implicit Processing at the ms Level.](http://www.ncbi.nlm.nih.gov/pubmed/26030155) *PLoS One* 10, e0127106. doi: 10.1371/journal.pone.0127106.
